# Pretreatment Staging FDG PET-Derived Radiomic Score Predicts Progression-Free Survival in Locally Advanced Rectal Cancer Treated with Total Neoadjuvant Therapy

**DOI:** 10.3390/diagnostics16142294

**Published:** 2026-07-22

**Authors:** Kadir Alper Kucuker, Onder Kaan Vezir, Aysegul Aksu, Adem Sengul, Ahmet Alacacioglu, Bulent Turgut

**Affiliations:** 1 Department of Nuclear Medicine, Izmir Katip Celebi University, 35360 Izmir, Turkey; onderkaanvezir@hotmail.com (O.K.V.); aaysegulgedikli@gmail.com (A.A.); bulturgut@yahoo.com (B.T.); 2 Department of Radiation Oncology, Izmir Katip Celebi University, 35360 Izmir, Turkey; adem.sengul@ikcu.edu.tr; 3 Department of Medical Oncology, Izmir Katip Celebi University, 35360 Izmir, Turkey; ahmet.alacacioglu@ikcu.edu.tr

**Keywords:** FDG PET, radiomics, rectal cancer, total neoadjuvant therapy, survival

## Abstract

**Background/Objectives**: Clinical outcomes in locally advanced rectal cancer (LARC) treated with total neoadjuvant therapy (TNT) remain heterogeneous, and available clinicopathological parameters provide limited prognostic accuracy. This study aimed to develop a radiomic score (RadScore) from baseline staging 18F-FDG PET/CT images using LASSO-penalized Cox regression, and to evaluate its ability to independently predict progression-free survival (PFS) in LARC patients treated with TNT followed by surgery. **Methods**: Eighty-five patients with LARC who underwent surgical resection after TNT between May 2018 and December 2024 were retrospectively analyzed. Primary tumor volumes were semi-automatically segmented on pretreatment FDG PET/CT images, and 140 radiomic features were extracted in accordance with Image Biomarker Standardisation Initiative (IBSI) guidelines. After correlation-based filtering, 23 non-redundant features were subjected to five-fold cross-validated LASSO-Cox regression for feature selection and RadScore construction. Internal validation used 1000-repetition bootstrap resampling. **Results**: Disease progression was recorded in 33 of 85 patients (mean PFS: 57.5 months). The final RadScore comprised two features—Intensity Histogram Kurtosis and GLCM Difference Entropy—and was identified as an independent prognostic factor in multivariable Cox analysis (HR = 1.81; 95% CI: 1.23–2.67; *p* = 0.003). The model achieved a C-index of 0.674, with a bootstrap-corrected C-index of 0.650 (95% CI: 0.568–0.741). Kaplan–Meier analysis demonstrated significantly shorter PFS in the high-risk group (log-rank *p* = 0.006). **Conclusions**: A pretreatment FDG PET-derived RadScore independently predicts PFS in LARC patients treated within a TNT framework, offering preliminary evidence for a potential imaging biomarker for preoperative risk stratification and individualized treatment planning—to our knowledge, among the first such evidence in a TNT-treated rectal cancer cohort.

## 1. Introduction

Rectal cancer accounts for approximately 30% of all colorectal cancer cases, which collectively represent the third most common malignancy and the second leading cause of cancer-related death worldwide, with nearly 1.9 million new cases and 904,000 deaths estimated in 2022 [1]. Its pelvic anatomy imposes unique challenges for resection and local control, and accurate pretreatment staging is essential for individualized treatment planning. While pelvic MRI remains the cornerstone of locoregional staging, 18F-fluorodeoxyglucose positron emission tomography/computed tomography (18F-FDG PET/CT) provides complementary whole-body metabolic imaging that can detect unsuspected distant metastases and nodal disease, thereby altering management in a clinically meaningful proportion of patients. Beyond staging, 18F-FDG PET/CT captures metabolic tumor burden through parameters such as maximum standardized uptake value (SUVmax), metabolic tumor volume (MTV), and total lesion glycolysis (TLG), and has demonstrated value in treatment response assessment and recurrence surveillance [2,3].

Locally advanced rectal cancer (LARC) that encompasses T3–T4 or node-positive disease is managed with a multimodal approach in which neoadjuvant chemoradiotherapy (nCRT) plays a central role. Standard long-course nCRT with concurrent fluoropyrimidine-based chemotherapy and 45–50.4 Gy pelvic radiotherapy followed by total mesorectal excision has been the established standard of care since the landmark German Rectal Cancer Study Group trial [4]. More recently, the paradigm has shifted toward total neoadjuvant therapy (TNT), integrating systemic chemotherapy into the preoperative phase, following the randomized controlled trials, which demonstrated improved pathological complete response rates and reduced distant treatment failure [4]. Despite these advances, clinical outcomes remain heterogeneous: pathological complete response is achieved in only 15–30% of patients, and a significant subset experiences disease progression after surgery, highlighting the unmet need for reliable pretreatment prognostic tools to guide risk-adapted strategies.

Radiomics has emerged as a promising approach to characterize intratumoral heterogeneity beyond conventional visual assessment with the high-throughput extraction of quantitative features from medical images. 18F-FDG PET-based radiomics offers a complementary metabolic dimension, capturing heterogeneity in glucose uptake that may reflect tumor aggressiveness, hypoxia, and clonal diversity. Prior studies have demonstrated that PET-derived textural features, particularly grey-level co-occurrence matrix (GLCM) parameters, are independently associated with survival outcomes and pathological response in LARC, outperforming conventional metabolic indices in several cohorts [5,6].

Despite the growing body of PET radiomics literature in rectal and colorectal cancer, a critical appraisal of existing evidence reveals important methodological limitations that constrain the generalizability of published findings. The majority of available studies are retrospective, single-center analyses with limited sample sizes, and externally validated prognostic radiomic models specifically predicting long-term oncological outcomes remain exceptionally scarce. Systematic reviews of the field have highlighted substantial heterogeneity in image acquisition protocols, segmentation methodologies, feature extraction pipelines, and statistical approaches across studies, rendering cross-study comparisons unreliable and impeding clinical translation [7,8]. Furthermore, most prior work has focused on conventional chemoradiotherapy rather than TNT, leaving the prognostic landscape of PET radiomics in the emerging TNT setting largely unexplored. Conventional PET metrics such as SUVmax, MTV, and TLG capture only aggregate or peak metabolic burden without encoding the spatial distribution and textural complexity of intratumoral FDG uptake, which may explain their inconsistent prognostic performance across studies and their displacement by textural features in multivariable analyses [9,10]. The present study was designed with awareness of these limitations, adopting IBSI-compliant feature extraction, LASSO penalization, and bootstrap internal validation to maximize methodological rigor within the constraints of a single-center retrospective design; nevertheless, external validation remains an essential next step before any clinical implementation can be considered. To the best of our knowledge, no study has yet investigated the prognostic value of pretreatment FDG PET radiomics specifically in patients treated within a TNT framework which is a setting in which preoperative risk stratification may have particular implications for treatment intensification and individualized decision-making. The present study aimed to address this gap by developing a LASSO-penalized Cox regression-based radiomics score (RadScore) from baseline staging 18F-FDG PET/CT images and evaluating its ability to independently predict PFS in 85 patients with rectal cancer treated with TNT.

## 2. Materials and Methods

### 2.1. Study Design and Patient Population

This retrospective study included 85 patients with LARC treated with TNT at our institution between May 2018 and September 2022. Inclusion criteria were: (1) histologically confirmed rectal adenocarcinoma, (2) locally advanced disease (cT3–T4 or cN+), (3) completion of TNT followed by surgical resection, and (4) availability of pre-treatment staging 18F-FDG PET/CT images in the institutional archive. Patients were excluded if they had distant metastatic disease at the time of staging, inadequate image quality, incomplete follow-up data, or a history of more than one primary tumor.

### 2.2. 18F-FDG PET/CT Image Acquisition

All PET/CT examinations were performed on a Discovery 710 PET/CT scanner (GE Medical Systems, Waukesha, WI, USA) following a standardized institutional protocol. Patients fasted for at least four hours prior to imaging and blood glucose levels were confirmed to be below 150 mg/dL before radiotracer injection. Each patient received an intravenous injection of 18F-FDG at a mean activity of 0.1 mCi/kg (3.7 MBq/kg), and image acquisition was initiated 60 ± 10 min after injection. Whole-body imaging was performed from vertex to mid-thigh, covering 7–8 bed positions at 2 min per bed position. Images were reconstructed in three dimensions using standard iterative reconstruction protocols. CT transmission scans were acquired at 120 kVp and 300 mA. PET emission data were reconstructed using an ordered-subset expectation maximization (OSEM) algorithm with 3 iterations and 24 subsets. The reconstructed voxel size was 4 mm × 4 mm × 4 mm.

### 2.3. Tumor Segmentation and Radiomic Feature Extraction

**Figure 1:**
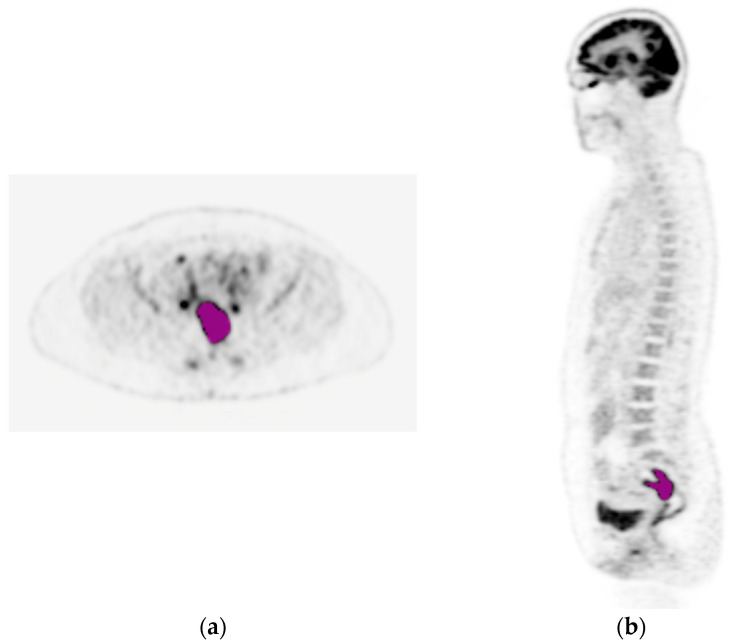
Segmentation of the primary tumor area in (**a**) axial and (**b**) sagittal sections.

Primary tumor volumes of interest (VOIs) were delineated on PET images using a semi-automated approach in LIFEx software (version 7.6.0) [11]. A nuclear medicine physician first manually outlined the tumor region on each transaxial slice, taking care to exclude adjacent areas of physiological FDG uptake, followed by automated refinement using an SUV threshold of 40% of the maximum voxel intensity within the delineated region. All segmentations were independently reviewed by a second nuclear medicine physician experienced in oncologic PET/CT interpretation; cases with visual disagreement regarding VOI boundaries were discussed until consensus was reached. Although formal interobserver variability analysis using intraclass correlation coefficients was not performed, the dual-reader consensus approach was adopted to minimize segmentation-related variability, consistent with published recommendations for PET radiomics studies (Figure 1) [12]. Prior to feature extraction, images were spatially resampled to an isotropic voxel size of 4 × 4 × 4 mm. Intensity values were preprocessed using absolute intensity rescaling to a fixed range of 0–40 SUV units and discretized into 64 grey levels with a bin size of 0.625, in accordance with IBSI guidelines, a total of 140 radiomic features were extracted from each VOI in compliance with the IBSI guidelines, encompassing morphological, intensity-based, intensity histogram, and texture feature categories—including GLCM, grey-level run-length matrix (GLRLM), neighbourhood grey-tone difference matrix (NGTDM), and grey-level size zone matrix (GLSZM) descriptors. All features were standardized using z-score normalization prior to modelling.

### 2.4. Feature Reduction and RadScore Development

To address multicollinearity, pairwise Spearman correlation filtering was applied with an absolute correlation threshold of |ρ| > 0.85, reducing the feature set from 140 to 23 candidates. A LASSO-penalized Cox proportional hazards regression model was then fitted to identify features independently associated with PFS, with the penalty parameter (lambda) optimized via five-fold cross-validation using the minimum partial likelihood deviance criterion (R software, version 4.6.0, glmnet package). Because the standard λ.1se criterion yielded an empty model in this relatively small cohort, the largest lambda value retaining at least one non-zero coefficient (λ.min-adjacent selection) was applied to ensure model parsimony while preserving prognostic signal. The final RadScore was computed as a linear combination of the selected features weighted by their LASSO-Cox coefficients.

### 2.5. Outcome Definition and Statistical Analysis

Progression-free survival was defined as the time from initial 18F-FDG PET/CT imaging to radiological or clinical disease progression or death from any cause; patients without an event at last follow-up were censored. The discriminative performance of the RadScore was assessed using Harrell’s concordance index (C-index). Internal validation was performed with 1000-repetition bootstrap resampling, yielding an optimism-corrected C-index with 95% confidence intervals. To evaluate clinical independence, a multivariable Cox regression model was constructed incorporating the RadScore alongside established clinicopathological prognostic variables, including perineural invasion (PNI), lymphovascular invasion (LVI), MTV, TLG, and SUVmax. The proportional hazards assumption was verified using Schoenfeld residuals. Patients were stratified into low- and high-risk groups using the optimal RadScore cutoff determined by the MaxStat method (maximizing the log-rank statistic), and Kaplan–Meier survival curves were compared using the log-rank test. All analyses were performed in R software (survival, glmnet, and Hmisc packages); a two-sided *p*-value < 0.05 was considered statistically significant.

### 2.6. Interobserver Reproducibility

Interobserver reproducibility of the radiomic features retained after correlation-based filtering was assessed in a randomly selected subset of 17 patients (20% of the cohort), in whom tumor segmentation was independently performed by a second nuclear medicine physician blinded to the first reader’s contours. Intraclass correlation coefficients (ICC; two-way random effects model, absolute agreement, single rater; ICC2,1) with 95% confidence intervals were calculated for all 23 features. The two features constituting the final RadScore demonstrated good interobserver reproducibility (ICC = 0.833 [95% CI: 0.602–0.936] and ICC = 0.814 [95% CI: 0.540–0.930], respectively; both *p* < 0.001). To further ensure the reproducibility of the radiomic features entering the modelling pipeline, only features demonstrating good-to-excellent interobserver reproducibility (ICC ≥ 0.75) were retained for subsequent LASSO-Cox regression.

## 3. Results

### 3.1. Patient Characteristics and Survival Summary

A total of 85 patients with locally advanced rectal cancer who underwent TNT were included. Disease progression was recorded in 33 patients during follow-up. The mean PFS was 57.5 months (95% CI: 48.5–66.4). Given the relatively high censoring rate, a reliable median PFS estimate could not be determined for the overall cohort (Table 1).

### 3.2. Feature Reduction and RadScore Construction

After application of the low-variance filter and Spearman correlation-based filtering (|ρ| > 0.85), the initial pool of 140 radiomic features was reduced to 23 non-redundant candidates (Supplementary Table S1). Of the 23 correlation-filtered features, 17 met ICC ≥ 0.75 threshold and were carried forward for feature selection; the remaining 6 features were excluded due to insufficient interobserver agreement. LASSO-penalized Cox regression identified two features as the most prognostically informative: Intensity Histogram Kurtosis (IBSI: C3I7) and GLCM Difference Entropy (IBSI: NTRS). Because the standard λ.1se criterion yielded an empty model owing to the limited sample size, the most parsimonious lambda retaining at least one predictor was selected. The final RadScore was derived from the corresponding LASSO-Cox coefficients as follows:

RadScore = (−0.345 × Intensity Histogram Kurtosis) + (−0.412 × GLCM Difference Entropy)

Both coefficients are negative, indicating that lower values of each feature reflecting greater intratumoral heterogeneity in pixel intensity distribution and local grey-level randomness, respectively, are associated with a higher RadScore and, consequently, with worse prognosis. Feature selection dynamics are illustrated in the LASSO coefficient path and cross-validation deviance plots.

### 3.3. Multivariable Cox Regression Analysis

In the multivariable Cox proportional hazards model including RadScore, PNI and LVI emerged as an independent prognostic factor for PFS (HR = 1.81; 95% CI: 1.23–2.67; *p* = 0.0026). Each one standard deviation increase in RadScore was associated with an approximately 81% increase in the risk of disease progression. PNI showed borderline significance in the same model (*p* = 0.057), while LVI did not reach statistical significance (*p* = 0.442). The proportional hazards assumption was verified using Schoenfeld residuals. Model fit was supported by a likelihood ratio test *p*-value of 0.003 (Table 2).

### 3.4. Risk Stratification and Kaplan–Meier Analysis

**Figure 2:**
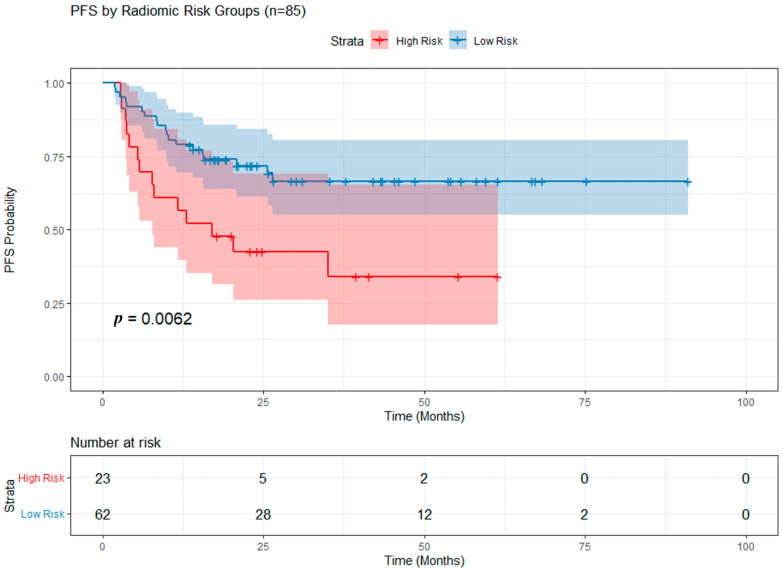
Kaplan–Meier survival curves showing the progression-free survival (PFS) stratified by radiomic risk groups (High-risk vs. Low-risk).

Using the optimal RadScore cutoff determined by the MaxStat method, patients were stratified into low-risk and high-risk groups. Kaplan–Meier analysis demonstrated a statistically significant difference in PFS between the two groups (log-rank *p* = 0.0062), with the high-risk group exhibiting markedly shorter progression-free survival (Figure 2).

### 3.5. Internal Validation

The RadScore model demonstrated an overall discriminative performance of C-index = 0.674 (SE = 0.045). Bootstrap internal validation (1000 iterations) yielded an optimism estimate of 0.025, resulting in an optimism-corrected C-index of 0.650 (95% CI: 0.568–0.741). These findings indicate moderate-to-good predictive performance and support the internal validity of the model.

### 3.6. Comparison of RadScore with Standard PET Parameters

To evaluate the prognostic superiority of the radiomic RadScore, univariable Cox regression analysis and bootstrap C-index calculation were performed for standard PET/CT volumetric and metabolic parameters (MTV, TLG, and SUVmax) in the same cohort. In univariable Cox analysis, MTV (HR = 1.159; 95% CI: 0.880–1.526; *p* = 0.294) and TLG (HR = 1.082; 95% CI: 0.785–1.491; *p* = 0.631) were not statistically significant predictors of progression risk. SUVmax similarly failed to reach statistical significance (HR = 0.805; 95% CI: 0.560–1.158; *p* = 0.242). In terms of discriminative performance, the C-index values of standard PET parameters remained close to chance level: 0.516 (95% CI: 0.410–0.621) for MTV, 0.527 (95% CI: 0.427–0.632) for TLG, and 0.567 (95% CI: 0.484–0.668) for SUVmax. These values were substantially lower than the bootstrap-corrected C-index achieved by the RadScore (0.622; 95% CI: 0.522–0.716), further supporting the added prognostic value of the radiomic model over conventional metabolic PET indices alone.

## 4. Discussion

To the best of our knowledge, this represents one of the first studies to develop and validate a PET-based radiomic prognostic model specifically in a cohort treated within a TNT framework, where early identification of patients at high risk of progression prior to any therapeutic intervention may directly inform decisions regarding treatment intensification, surveillance intensity, and adjuvant therapy planning. In this retrospective study of 85 patients with locally advanced rectal cancer treated with TNT, we developed a two-feature FDG PET/CT-derived radiomic score comprising Intensity Histogram Kurtosis and GLCM Difference Entropy that independently predicted progression-free survival in multivariable Cox analysis (HR = 1.81; 95% CI: 1.23–2.67; *p* = 0.003), retaining its prognostic significance after adjustment for perineural invasion, lymphovascular invasion, and conventional metabolic parameters including SUVmax, MTV, and TLG. Bootstrap internal validation yielded a corrected C-index of 0.650, supporting moderate-to-good discriminative performance. These findings indicate that intratumoral metabolic heterogeneity, as captured by textural and histogram-based PET features at the time of initial staging, encodes prognostic information that is not reflected by standard imaging metrics or established clinicopathological variables, and may contribute to individualized risk stratification prior to treatment initiation. In the present study, the initial number of radiomic features was reduced using a two-step feature selection approach. First, highly correlated features were filtered to avoid redundancy and minimize the inclusion of overlapping information in the model. Subsequently, LASSO-Cox regression was applied to the remaining features, and two were retained in the final model. Therefore, these two features should be interpreted as the radiomic parameters that provided the most relevant prognostic contribution within our dataset, rather than as markers selected solely on the basis of biological interpretation.

Intensity Histogram Kurtosis (the normalized fourth central moment of the intratumoral SUV distribution) quantifies the peakedness of the uptake histogram relative to a normal distribution. A lower kurtosis value indicates a flatter, more dispersed intensity distribution, reflecting greater variability in metabolic activity across voxels and implying a more heterogeneous tumor microenvironment. In our model, lower kurtosis was associated with higher RadScore and worse prognosis, consistent with the established relationship between intratumoral metabolic heterogeneity and aggressive tumor biology. Kurtosis has been reported as a prognostic histogram feature across different malignancies: it was identified as a component of a radiomics signature predicting disease-free survival in lung cancer [13], and intensity-based kurtosis from pretreatment FDG PET was significantly associated with overall survival in advanced high-grade serous ovarian cancer [14]. Kurtosis and related histogram moments have also been proposed as markers of intratumoral spatial heterogeneity in cervical cancer treated with radiotherapy [15]. GLCM Difference Entropy, the second component of the RadScore, quantifies the randomness of grey-level differences between spatially adjacent voxels; higher values reflect greater local textural disorder and a more disorganized intratumoral uptake pattern. In our cohort, lower Difference Entropy was associated with worse prognosis. The inverse association between lower GLCM Difference Entropy and worse prognosis warrants careful consideration. GLCM Difference Entropy is mathematically defined as the entropy of the probability distribution of absolute grey-level differences between co-occurring voxel pairs—that is, it quantifies the randomness of local intensity transitions rather than the overall distribution of intensity values within the tumor. This distinguishes it fundamentally from joint entropy or first-order histogram entropy, which measure the unpredictability of the global SUV distribution. A tumor with low GLCM Difference Entropy therefore exhibits predictable, spatially structured local intensity transitions—not necessarily a homogeneous uptake pattern—which may paradoxically coexist with a highly organized but biologically aggressive clonal architecture. From a biological standpoint, this pattern may reflect tumors in which metabolically active subregions are spatially compartmentalized and structured, rather than randomly interdigitated—a configuration associated with dominant aggressive clones occupying discrete spatial niches rather than diffuse heterogeneous infiltration. Such structured metabolic compartmentalization has been linked to higher proliferative capacity and resistance to cytotoxic therapy in solid tumors, as spatially organized high-grade clones may be less susceptible to the heterogeneous cytotoxic pressure of chemoradiotherapy. The prognostic relevance of GLCM entropy-family features in FDG PET radiomics of rectal cancer is well supported: Hotta et al. reported that GLCM entropy was an independent prognostic factor for both PFS and overall survival in 94 surgically treated rectal cancer patients, outperforming conventional PET parameters in multivariable Cox regression [16]. Similarly, Amrane et al. demonstrated that Histogram Entropy remained an independent predictor of relapse-free and overall survival in gastro-esophageal junction cancer, with SUVmean and volumetric parameters failing to reach independent significance [17]. Importantly, neither PNI nor LVI retained independent prognostic significance in our model, suggesting that pretreatment imaging-derived metabolic heterogeneity may encode tumor biological information that is complementary to postoperative pathological features, which represent focal sampling of a spatially heterogeneous lesion at a single time point.

The bootstrap-corrected C-index of 0.650 is consistent with the performance range reported in comparable single-center PET radiomics studies. Lovinfosse et al., in 86 LARC patients, demonstrated that pretreatment FDG PET/CT texture parameters independently predicted disease-free survival, while SUVmax, MTV, and TLG failed to reach independent significance [9]. Hotta et al. reported analogous findings in surgically treated rectal cancer [16]. PET radiomics models in broader gastrointestinal and gynecological cancer cohorts have yielded C-indices in the range of 0.69–0.71 for PFS prediction [18], placing our two-feature internally validated model at the lower end of this range—an expected finding given the limited sample size and absence of external validation. The failure of SUVmax, MTV, and TLG to independently predict PFS in our model is consistent with a broader pattern across LARC studies, in which conventional baseline PET parameters are displaced by textural features in multivariable analysis [14], and at least one study found no significant association between any standard PET metric and long-term survival after nCRT [19]. These observations suggest that point-wise or aggregate metabolic measurements capture only a limited dimension of tumor biology without encoding the spatial heterogeneity of intratumoral FDG uptake.

The RadScore has potential relevance as a practical decision-support tool at the time of initial staging. Because it is derived entirely from the pretreatment FDG PET/CT scan its implementation would not require additional imaging or invasive procedures. In the current era of TNT and individualized treatment escalation, should these findings be confirmed in prospective multicenter studies, patients identified as high-risk could be considered for intensified neoadjuvant regimens or closer postoperative surveillance, while low-risk patients might be spared unnecessary treatment intensification, in line with the growing emphasis on organ preservation in LARC management. Clinical translation will require, however, not only external multicenter validation but also standardization of image acquisition, segmentation, and feature extraction pipelines [20]. Integration of the RadScore with complementary biomarkers including ctDNA, molecular tumor profiling, and MRI-based radiomics may further enhance risk stratification beyond what any single modality can achieve [21].

Several limitations of this study should be acknowledged. First, the retrospective single-center design introduces inherent selection bias and limits the generalizability of the findings. Second, the relatively small sample size of 85 patients constrains the statistical power of the multivariable model and increases the risk of overfitting, despite the application of LASSO penalization and bootstrap internal validation. Third, external validation in an independent cohort was not performed; consequently, the prognostic performance of the RadScore reported here reflects internal validity only and requires prospective multicenter confirmation before clinical translation. Fourth, PET/CT image acquisition was performed on a single scanner model using a standardized institutional protocol, which, while ensuring intra-study consistency, may limit the reproducibility of radiomic features across different scanners, reconstruction algorithms, and acquisition parameters—a recognized challenge in multicenter radiomics research. Fifth, tumor segmentation was performed semi-automatically with SUV-based thresholding and dual-reader consensus; although this approach reduces inter-observer variability, fully automated segmentation pipelines would further enhance reproducibility. Sixth, the optimal RadScore cutoff was determined using the MaxStat method on the same dataset used for model development, which may introduce an optimistic bias in the Kaplan–Meier risk group comparison despite bootstrap correction of the C-index. Seventh, the multivariable model included perineural invasion and lymphovascular invasion as covariates but did not incorporate other potentially relevant prognostic variables such as pathological tumor regression grade, circumferential resection margin status, or molecular markers, due to incomplete data availability in this retrospective cohort. Finally, the relatively high censoring rate precluded reliable estimation of median PFS for the overall cohort, and longer follow-up will be needed to assess the RadScore’s association with overall survival.

## 5. Conclusions

In this retrospective single-center study, a pretreatment 18F-FDG PET/CT-derived two-feature radiomic score—incorporating Intensity Histogram Kurtosis and GLCM Difference Entropy—independently predicted progression-free survival in patients with locally advanced rectal cancer treated with TNT. RadScore retained its prognostic significance after adjustment for established clinicopathological variables and conventional PET parameters and demonstrated moderate-to-good discriminative performance on internal validation. These findings provide preliminary evidence that pretreatment intratumoral metabolic heterogeneity, as quantified by PET-derived textural features at the time of initial staging, encodes biologically meaningful prognostic information that is complementary to standard clinical and imaging metrics. Prospective multicenter validation is warranted to establish the generalizability and clinical utility of the RadScore as a preoperative risk stratification tool in locally advanced rectal cancer.

## Figures and Tables

**Table 1:** Demographic and clinicopathological features of the patients.

Feature		No (%)
Sex	Male Female	55 (64.7) 30 (35.3)
Age	Mean	59 ± 11
Clinical Stage	Stage II Stage III	39 (45.8) 46 (54.2)
Lymphovascular invasion	Positive Negative	31 (36.5) 54 (63.5)
Perineural invasion	Positive Negative	18 (21.2) 67 (78.8)
Progression in follow-up	Positive Negative	33 (38.8) 52 (61.2)
Pathologic Response of Primary Tumor	0 1+ 2+ 3+	13 (15.3) 10 (11.8) 37 (43.5) 25 (29.4)

**Table 2:** Variables of Multivariable Cox Regression Analysis.

Variable	Beta	HR	95% CI	*p*-Value
RadScore	0.595	1.81	1.23–2.67	0.003 *
PNI (positive)	0.851	2.34	0.97–5.63	0.057
LVI (positive)	0.315	1.37	0.61–3.06	0.442

* Statistically significant.

## Data Availability

The data presented in this study are not publicly available due to privacy and ethical restrictions related to patient clinical and imaging data. De-identified data may be made available from the corresponding author upon reasonable request and subject to institutional and ethical approval.

## Supplementary Materials

The following supporting information can be downloaded at: https://www.mdpi.com/article/10.3390/diagnostics16142294/s1, Supplementary Table S1: Radiomic Features Retained After Correlation-Based Filtering and Their Definitions.

## Abbreviations

The following abbreviations are used in this manuscript:

18F-FDG	Fluorine-18 fluorodeoxyglucose
PET/CT	Positron emission tomography/computed tomography
LARC	Locally advanced rectal cancer
TNT	Total neoadjuvant therapy
nCRT	Neoadjuvant chemoradiotherapy
PFS	Progression-free survival
RadScore	Radiomic score
LASSO	Least absolute shrinkage and selection operator
IBSI	Image Biomarker Standardisation Initiative
MRI	Magnetic resonance imaging
SUV	Standardized uptake value
SUVmax	Maximum standardized uptake value
MTV	Metabolic tumor volume
TLG	Total lesion glycolysis
VOI	Volume of interest
GLCM	Grey-level co-occurrence matrix
GLRLM	Grey-level run-length matrix
NGTDM	Neighbourhood grey-tone difference matrix
GLSZM	Grey-level size zone matrix
PNI	Perineural invasion
LVI	Lymphovascular invasion
HR	Hazard ratio
CI	Confidence interval
C-index	Concordance index
SE	Standard error
ctDNA	Circulating tumor DNA
TNM	Tumor–node–metastasis
Gy	Gray
MBq	Megabecquerel
mCi	Millicurie
