# Supplementary material for: Pretreatment Staging FDG PET-Derived Radiomic Score Predicts Progression-Free Survival in Locally Advanced Rectal Cancer Treated with Total Neoadjuvant Therapy
Source: Diagnostics (Basel). 2026 Jul 22;16(14):2294. doi: 10.3390/diagnostics16142294 (PMC13407901; doi:10.3390/diagnostics16142294)
Supplement: Supplementary file 1 [file diagnostics-16-02294-s001.zip › diagnostics-4384143-supplementary.pdf]

Supplementary Tables

Supplementary Table S1. Radiomic Features Retained After Correlation-Based Filtering and Their Definitions.

No	Feature Name	IBSI Code	Feature Class	Definition
1	Surface-to-Volume Ratio	2PR5	Morphological	Ratio of the tumor surface area to its volume. Higher values indicate a more irregular, complex tumor shape with greater surface exposure relative to its size.
2	Sphericity	QCFX	Morphological	Measure of how closely the tumor shape approximates a perfect sphere, ranging from 0 to 1. Values approaching 1 indicate a more spherical and regular tumor morphology.
3	Max Intensity Coordinate—ROI Centroid Distance	—	Morphological	Euclidean distance between the voxel of maximum SUV intensity and the geometric centroid of the tumor volume. Reflects the spatial eccentricity of the hottest metabolic focus within the lesion.
4	Radius ROI Norm—Max Intensity–Centroid Distance	—	Morphological	Normalized version of feature 3, expressing the max-intensity-to-centroid distance relative to the equivalent sphere radius of the ROI. Facilitates size-independent shape comparison.
5	Radius Sphere Norm—Centroid–Weighted Centroid Distance	—	Morphological	Normalized distance between the geometric centroid and the intensity-weighted centroid (center of mass) of the tumor. Quantifies asymmetry between the morphological and metabolic centers of the lesion.
6	Radius Sphere Norm—Max Intensity–Perimeter Distance (2D Coronal)	—	Morphological	Normalized distance from the maximum-intensity voxel to the nearest tumor boundary in the coronal plane. Reflects how centrally or peripherally the hottest metabolic region is located within the tumor in the coronal orientation.
7	Radius Sphere Norm—Max Intensity–Perimeter Distance (2D Sagittal)	—	Morphological	Same as feature 6 measured in the sagittal plane. Together with feature 6, provides a multiplanar assessment of the spatial relationship between peak metabolic activity and the tumor margin.
8	Minimum Intensity	1GSF	Intensity-based	Lowest SUV value within the tumor VOI. Sensitive to the presence of necrotic or hypometabolic subregions, which may reflect intratumoral heterogeneity or areas of treatment-related change.
9	Intensity-Based Coefficient of Variation	7TET	Intensity-based	Standard deviation of SUV values divided by their mean within the VOI. A dimensionless measure of relative SUV dispersion; higher values indicate greater intratumoral metabolic variability.
10	Intensity-Based Quartile Coefficient of Dispersion	9S40	Intensity-based	Interquartile range of SUV values divided by their sum of the first and third quartiles. A robust, outlier-resistant measure of SUV spread within the tumor.
11	Intensity Histogram Skewness	88K1	Intensity histogram	Third standardized central moment of the intratumoral SUV distribution. Measures the asymmetry of the histogram; positive skewness indicates a tail toward higher SUV values, suggesting a small proportion of highly metabolically active voxels.
12	Intensity Histogram Kurtosis	C3I7	Intensity histogram	Fourth standardized central moment of the SUV distribution. Quantifies the "peakedness" of the histogram relative to a normal distribution. Lower kurtosis (platykurtic) reflects a flatter, more dispersed uptake distribution, indicative of greater intratumoral metabolic heterogeneity.
13	Intensity Histogram Mode	AMMC	Intensity histogram	Most frequently occurring SUV value within the tumor histogram. Represents the dominant metabolic activity level across tumor voxels; may differ substantially from the mean in heterogeneous tumors.
14	Maximum Histogram Gradient Grey Level	8E6O	Intensity histogram	SUV value at which the histogram shows the steepest upward slope. Reflects the grey level at the transition from low to high voxel frequency, capturing the rising edge of the metabolic distribution.
15	Minimum Histogram Gradient Grey Level	RHQZ	Intensity histogram	SUV value at which the histogram shows the steepest downward slope. Captures the falling edge of the distribution, providing complementary information about the upper tail of metabolic activity.

16	GLCM Joint Maximum	GYBY	GLCM	Highest value in the grey-level co-occurrence matrix, representing the most frequently co-occurring SUV pair between adjacent voxels. High values suggest spatial uniformity in local uptake patterns.
17	GLCM Difference Entropy	NTRS	GLCM	Entropy of the difference distribution between grey levels of co-occurring voxel pairs. Quantifies the randomness or unpredictability of local SUV differences; higher values indicate greater textural disorder and spatial heterogeneity of metabolic uptake.
18	GLCM Normalised Inverse Difference Moment	1QCO	GLCM	Measures local homogeneity of the SUV texture, normalized to reduce sensitivity to large grey-level differences. Values close to 1 indicate a spatially homogeneous uptake pattern with small variations between adjacent voxels.
19	GLCM Correlation	NI2N	GLCM	Measures the linear dependency of grey-level values between co-occurring voxel pairs. High correlation reflects a structured, spatially predictable uptake pattern; low correlation indicates disorganized heterogeneity.
20	GLCM Cluster Shade	7NFM	GLCM	Reflects the skewness and asymmetry of co-occurrence clusters within the texture matrix. High absolute values indicate pronounced asymmetric clustering in the local uptake pattern, associated with perceptually complex textures.
21	GLRLM Run Length Non-Uniformity	W92Y	GLRLM	Measures variability in the lengths of consecutive voxels sharing the same grey level along a given direction. Lower values indicate more uniform run lengths (homogeneous texture); higher values reflect irregular, non-uniform spatial patterns of uptake.
22	NGTDM Busyness	NQ30	NGTDM	Ratio of grey-level changes between a voxel and its neighbours to the total number of voxel-neighbour pairs in the neighbourhood grey-tone difference matrix. High busyness reflects rapid, frequent spatial transitions in SUV, indicating a highly heterogeneous uptake texture.
23	GLSZM Large Zone High Grey Level Emphasis	J17V	GLSZM	Measures the joint occurrence of large connected regions of homogeneous uptake with high grey-level (SUV) values. High values suggest the presence of extensive metabolically active zones, potentially reflecting bulky, high-uptake tumor subregions.

Abbreviations: IBSI, Image Biomarker Standardisation Initiative; SUV, standardized uptake value; VOI, volume of interest; ROI, region of interest; GLCM, grey-level co-occurrence matrix; GLRLM, grey-level run-length matrix; NGTDM, neighbourhood grey-tone difference matrix; GLSZM, grey-level size zone matrix; 2D, two-dimensional.
